# VPAC1 and VPAC2 receptors mediate tactile hindpaw hypersensitivity and carotid artery dilatation induced by PACAP38 in a migraine relevant mouse model

**DOI:** 10.1186/s10194-024-01830-2

**Published:** 2024-07-31

**Authors:** Song Guo, Rikke Holm Rasmussen, Anders Hay-Schmidt, Messoud Ashina, Ayodeji A. Asuni, Jeppe Møller Jensen, Anja Holm, Sabrina Prehn Lauritzen, Glenn Dorsam, Jens Hannibal, Birgitte Georg, David Møbjerg Kristensen, Jes Olesen, Sarah Louise Christensen

**Affiliations:** 1 Department of Neurology, Danish Headache Center, Copenhagen University Hospital – Rigshospitalet Glostrup, Copenhagen, Denmark; 2 Translational Research Centre (TRACE), Copenhagen University Hospital, Rigshospitalet, Glostrup, Denmark; 3 Department of Odontology, Panum Institute, Faculty of Health, University of Copenhagen, Copenhagen, Denmark; 4 Department of Clinical Medicine, Faculty of Health and Medical Sciences, University of Copenhagen, Copenhagen, Denmark; 5 Department of Preclinical Fluid Biomarkers and Occupancy, H. Lundbeck A/S, Copenhagen, Denmark; 6 Center for RNA Medicine, Department of Clinical Medicine, Aalborg University, Copenhagen, Denmark; 7 Department of Experimental Clinical Research, Translational Research Centre, Rigshospitalet Glostrup, Copenhagen, Denmark; 8 Department of Microbiological Sciences, North Dakota State University, Fargo, USA; 9 Department of Clinical Biochemistry, Copenhagen University Hospital - Bispebjerg and Frederiksberg, Copenhagen, Denmark; 10 Department of Growth and Reproduction, Copenhagen University Hospital – Rigshospitalet, Copenhagen, Denmark; 11 Department of Science and Environment, Roskilde University, Roskilde, Denmark; 12 Department of Anesthesia, Critical Care and Pain Medicine, Beth Israel Deaconess Medical Center, Harvard Medical School, Boston, MA USA

**Keywords:** PACAP, VPAC1, VPAC2, PAC1, Migraine, von Frey, Knockout mice

## Abstract

**Background:**

Pituitary adenylate cyclase-activating peptide (PACAP) is a neuropeptide pivotal in migraine pathophysiology and is considered a promising new migraine drug target. Although intravenous PACAP triggers migraine attacks and a recent phase II trial with a PACAP-inhibiting antibody showed efficacy in migraine prevention, targeting the PACAP receptor PAC1 alone has been unsuccessful. The present study investigated the role of three PACAP receptors (PAC1, VPAC1 and VPAC2) in inducing migraine-relevant hypersensitivity in mice.

**Methods:**

Hindpaw hypersensitivity was induced by repeated PACAP38 injections. Tactile sensitivity responses were quantified using von Frey filaments in three knockout (KO) mouse strains, each lacking one of the PACAP-receptors (N_total_ = 160). Additionally, ex vivo wire myography was used to assess vasoactivity of the carotid artery, and gene expression of PACAP receptors was examined by qPCR.

**Results:**

PACAP38 induced hypersensitivity in WT controls (*p* < 0.01) that was diminished in VPAC1 and VPAC2 KO mice (*p* < 0.05). In contrast, PAC1 KO mice showed similar responses to WT controls (*p* > 0.05). Myograph experiments supported these findings showing diminished vasoactivity in VPAC1 and VPAC2 KO mice. We found no upregulation of the non-modified PACAP receptors in KO mice.

**Conclusions:**

This study assessed all three PACAP receptors in a migraine mouse model and suggests a significant role of VPAC receptors in migraine pathophysiology. The lack of hypersensitivity reduction in PAC1 KO mice suggests the involvement of other PACAP receptors or compensatory mechanisms. The results indicate that targeting only individual PACAP receptors may not be an effective migraine treatment.

## Introduction

Migraine affects over 1 billion people worldwide and ranks as the second leading cause of years lived with disability [1] Over the last decade, human and animal provocation models have shed light on the pathophysiology of migraine. Notably, two endogenous neuropeptides, calcitonin gene-related peptide (CGRP) and pituitary adenylate cyclase-activating peptide (PACAP), that induce dilation of cephalic arteries when administrated intravenously in humans, have been identified as triggers of migraine attacks [2, 3] This research has contributed to the development of CGRP-targeted therapies, providing a major therapeutic breakthrough for many migraine sufferers worldwide [4] However, some data suggest that approximately 40% of adults with migraine do not benefit from these new treatments [5] PACAP may be an important player in these patients as PACAP acts via a pathway distinct from the CGRP pathway in rodent models of migraine [6] In addition, a PACAP targeted monoclonal antibody (mAb) was effective in migraine prevention in a recent phase II trial, whereas targeting the PACAP receptor PAC1 alone has been unsuccessful [7].

PACAP, a multi-functional peptide, shares receptors with other peptides, such as the vasoactive intestinal peptide (VIP), which shares 68% sequence homology with PACAP [8] The three main PACAP receptors (VPAC1, VPAC2 and PAC1) are all found in the migraine-relevant trigeminovascular system e.g. the trigeminal ganglia, sphenopalatine ganglia, and cranial arteries [9] VPAC1 and VPAC2 receptors have equal affinity for PACAP38 and VIP, while the PAC1 receptor has a much higher affinity for PACAP38 than for VIP [10]. VIP infusion triggers migraine attacks in adults with migraine and headache in healthy volunteers but only when the infusion is prolonged from 20 min to 2 h [11, 12]. A better understanding of the migraine signaling pathways associated with PACAP could facilitate the identification of new treatment targets.

The objective of the present study was to explore PACAP-induced hypersensitivity in a validated mechanistic mouse model of migraine using genetically modified mice for one or more of its three receptors. This is a unique and necessary approach given the cross binding of many of the commercially available pharmacological antagonists on these receptors. We also conducted ex vivo experiments using myographs to further substantiate vasoactivity and employed qPCR to investigate a potential compensatory upregulation of the non-modified PACAP receptors in KO mice.

## Materials and methods

### Experimental animals

PACAP receptor KO mice (for each receptor subtype VPAC1, VPAC2, and PAC1) and WT controls for each strain bred in-house were used for this study. In total 160 mice of 8–18 weeks of age with an equal sex distribution were used. The VPAC1 KO breeders were provided by Prof. Glenn Dorsam from North Dakota State University, USA and the VPAC2 and PAC1 KO breeders were provided by Prof. Jens Hannibal from Bispebjerg and Frederiksberg Hospitals, Denmark. The generation of VPAC1 (exon 4–6 deletion), VPAC2 (exon 8–10 deletion) and PAC1 (exon 8–11 deletion) KO mice were previously described [13–15]. The mice were cared for under the same conditions as previously published [16]. Groups of six mice were housed in Eurostandard type III cages (42.5 × 27.6 × 15.3 cm, Tecniplast, Italy) in a temperature and light controlled room (lights on at 07:00 with a 12 h light/dark cycle) with food and water ad libitum in cages with shelters and nesting material for enrichment purposes. The VPAC1 strain was difficult to breed, with increased pre-natal and post-natal mortality, as previously described [13]. However, delaying weaning until 6 weeks of age and food supplement using DietGel Boost from ClearH2O decreased mortality significantly. Mice weighed between 14 and 30 g, and as a general health assessment all mice were weighed on every test day. Mice in experiments were euthanized by intraperitoneal injection of a 100 µL mixture of 200 mg/mL pentobarbital and 20 mg/mL lidocaine (Glostrup Pharmacy) or by decapitation after carbon dioxide (mixed gas) inhalation. The experiments were conducted in accordance with ARRIVE guidelines and with the European Community guide for the care and use of animals (2010/63/UE) and were approved by the Danish Animal Experiments Inspectorate (ethical approval number 2022-15-0201-01347 and 2023-15-0202-00187).

### Experimental design and protocols

**Fig. 1:**
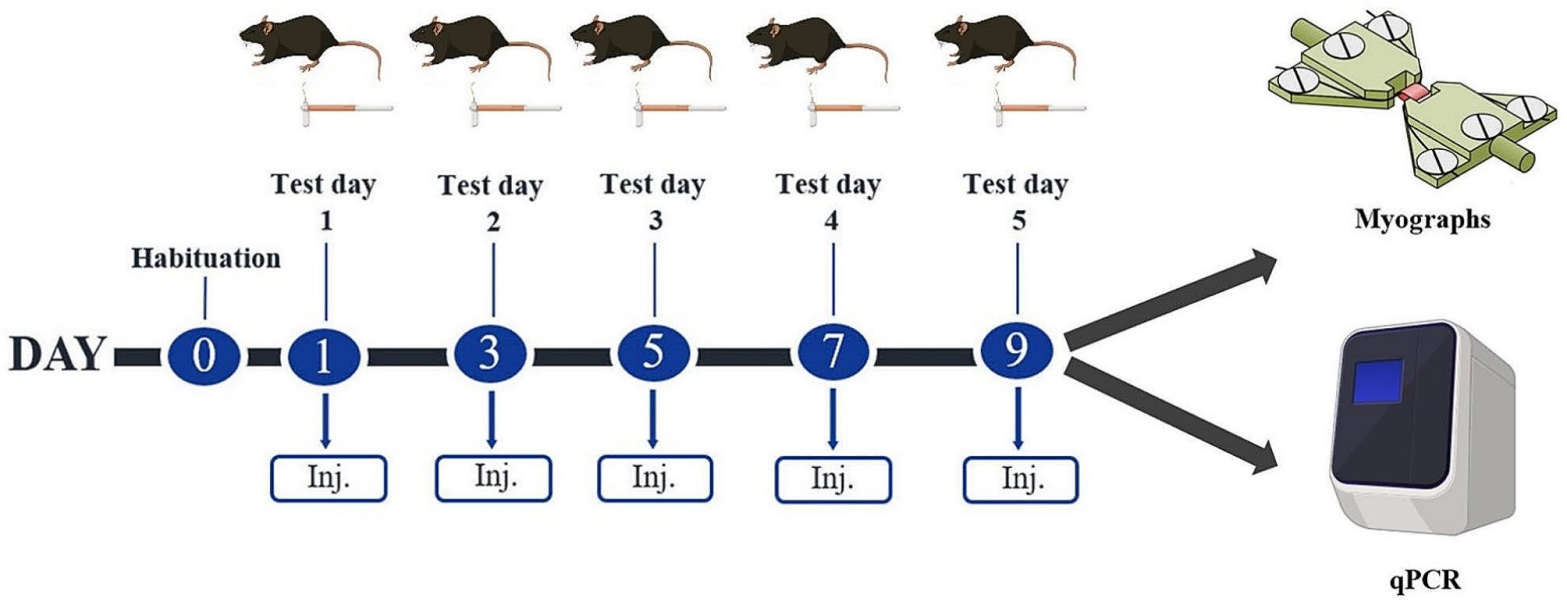
Design and experimental timeline of test paradigm for subcutaneous PACAP38 or saline injection in KO or WT mice. Following 1 day of habituation (day 0), five tests were done every other day over the course of 9 days. On every test day, the basal threshold of cutaneous sensitivity was measured using von Frey filaments prior to injections and the acute response was measured 1 h after PACAP38 injection. On day 9, tissues were sampled for qPCR. Carotid artery from saline treated mice were sampled between 1–4 weeks after day 9 for wire myography. Reproduced with modifications from reference [28]. Created with BioRender

We used an in-house validated mechanistic mouse model of migraine with PACAP38 peptide as the migraine-inducing agent [6, 17] and evaluated its effects by measurement of cutaneous sensitivity as previously described [18]. The PACAP38 model has been validated for its relevance to migraine by two different research groups [6, 19] and we followed the provocation and test protocol described in [6, 17]. In three independent experiments, PACAP38 was injected to KO mice for each of the three PACAP receptor subtypes (PAC1, VPAC1 and VPAC2) and their WT controls followed by sensory response measurements using von Frey filaments as a surrogate marker of migraine pain response [18]. Group size was 12 mice in the experiments except for VPAC2 KO mice which included 16 mice per group as VPAC2 KO mice were much easier to breed. Two male VPAC1 KO mice were found dead in their home cage on day 5 of the experiment. The data from these (both were saline controls) were excluded from the experiment resulting in this group ending up with only 10 mice. Mice of both sexes were tested every other day on 5 test days spanning over 9 days (days 1, 3, 5, 7, 9). On every test day, the basal threshold of cutaneous sensitivity was measured prior to injections (that is 48 h after last injection), and the acute response was measured 1 h after injection. All tests were conducted in low-light conditions (20–30 lx) in the timeframe of 8:00–15:00 by a blinded experimenter. On day 9 of the protocol, tissues were sampled for qPCR. Carotid artery samples for wire myography were from saline treated mice and sampled one to four weeks after the completion of the von Frey experiments. An overview of the study design is provided in Fig. 1.

### Breeding and genotyping

The three KO mice strains (VPAC1, VPAC2, and PAC1) and WT controls were bred from heterozygous breeding pairs on C57BL/6 background. Original PAC1 KO breeders mice were on 129/SvXC57BL6/J background but were backcrossed with C57BL/6J BomTAC mice. Subsequent breeding for homozygous KO and WT animals were continuously monitored through genotyping using PCR analysis. DNA was extracted from ear punch samples. Protocol and primer design was based on previously described publications for the three strains [13–15].

### Behavioral tests

#### Cutaneous sensitivity to tactile stimulation

Cutaneous sensitivity was evaluated on the plantar region of the left hind paw using von Frey filaments (Ugo Basile, Italy), starting with the 0.16 g filament and using the range 0.008–2.0 g (excluding 1.4 g). For our assessment, we employed the up-down method [20]. The 50% withdrawal threshold in grams was calculated using the freely accessible online tool: https://bioapps.shinyapps.io/von_frey_app with exact δ and target force settings [21]. For the test, mice were placed in individual, transparent plexiglas chambers with mesh floor (IITC Life Science). To familiarize them with the testing environment, mice were allowed to acclimate in these chambers for 45 min before the first test (day 0). Additionally, before each subsequent test mice were placed in the chamber 30-minute prior to the test.

#### Motor function (rotarod)

To ensure that the cutaneous sensitivity test using von Frey filaments was not affected by potential motor impairments in KO mice, general motor function was assessed using a rotarod (Rotarods Advanced, IITC Life Science Inc.). The rotarod assessment was performed before the first PACAP38 injection and right after the last von Frey test on test day 9. Mice were given a single attempt on the rotarod. It started at 0 rpm, gradually increased to 30 rpm within 45 s, and ended after 150 s (the maximum duration).

### Test agents

PACAP38, manufactured by CASLO ApS (purity > 95%, 5% freeze-dried acetic acid, HPLC), Denmark, was injected subcutaneously (s.c.) in the loose skin between the abdomen and right hind leg using 25G BD MicrolanceTM needles from NJ, USA while the mouse was gently restrained by neck scuffing. The administered volume was 5 mL/kg. A prior in-house study determined the optimal dose to be 2 µg/kg [6] which was used for the injections. To achieve this concentration, PACAP38 was dissolved and diluted in saline to 0.4 µg/mL. Saline was injected as control.

### Myographs

We assessed the effect of PACAP38 on vasoactivity of carotid arteries from the three strains of KO mice using the ex vivo wire myograph method [22]. The distribution of KO mice and WT mice was balanced across sex, age, and day of the experiment. WT and KO mice were euthanized with 100 µL pentobarbital i.p., (combination preparation 200 mg/mL pentobarbital + 20 mg/mL lidocaine, Glostrup Pharmacy, Denmark). The distal part of the carotid artery was isolated and cut into segments of approximately 1 mm, then immersed in oxygenated Na^+^ Krebs buffer and mounted on 40 μm diameter wires in a Mulvany-Halpern wire myograph. The myograph baths are warmed to 37 °C and the vessels are left to settle for at least 15 min, before stretching the vessels to achieve a pretension of 2 mN/mm. Vessel contractility is then examined by changing the buffer oxygenated K^+^ Krebs. A precontraction is induced with 0.03 µM of the prostaglandin analog/thromboxane A2 agonist U46619, purchased from Tocris/Bio-Techne, UK, followed by administration of increasing concentrations of PACAP-38 (10^− 9^ M-10^− 6^ M). Following a new precontraction with 0.03 µM U46619, the endothelium function was evaluated by addition of increasing concentrations of carbachol (10^− 8^ M-10^− 6^ M) purchased from Sigma-Aldrich/Merck, Belgium. The relaxation induced by both PACAP-38 and carbachol was then calculated using the following equation:

$$100 - \left( {100 \cdot {\matrix{tension\;of\>the\;blood\;vessel\;after\;stimulation \hfill \cr \,\,\,\,\,\,\,\,\,\,\,\,\,\,\,\,\,\,\,\,\,\,\,\,\,\,\,\,\,\,\,\,\,\,\, - max\;relaxation\;potential \hfill \cr} \over {100\%\;precontraction - {\rm{max}}\;relation\;potential}}} \right)$$

where x is the tension of the blood vessel after stimulation. 100% precontraction and max relaxation potential are constants for individual blood vessels.

### qPCR

#### RNA isolation from tissues

Immediately after euthanasia, brainstem containing the trigeminal nucleus caudalis (TNC), trigeminal ganglion (TG), dura mater, and carotid arteries were dissected and stored at -80^o^C in 2 mL FastPrep Lysing Matrix tubes (MP Biomedicals) with ceramic beads [23]. Total RNA was isolated using spin columns (NucleoSpin miRNA, Machery Nagel) in combination with QIAzol (Qiagen) and chloroform (Sigma) according to the manufacturer’s recommendations (version 07-2013, rev. 03). The samples were homogenized using QIAzol lysis buffer (Qiagen) and 1.4 mm ceramic beads (Lysing Matrix D, MP Biomedicals, USA) for 40 s. at max speed using a FastPrep-24TM 5G instrument (MP Biomedicals, USA). The RNA concentration was measured using a Nanodrop 2000c (Thermofisher) at 260 nm.

#### cDNA synthesis and quantitative real-time PCR

1 µg RNA was reverse transcribed using iScript cDNA Synthesis kit (Bio-Rad) according to the recommendation of manufacture in a total volume of 20 µL. The qPCR was performed using pre-designed TaqMan mRNA assay (Table 1) and TaqMan PCR Master Mix from Integrated DNA Technologies (Iowa, USA) in a 10 µl reaction volume using the QuantStudio 6 Pro Real-Time PCR instrument (Applied Biosystems by Thermo Fischer Scientific, Foster City, CA, USA). The thermal cycling condition for qPCR amplification included: 2 min at 50ºC; 10 min at 95ºC; 45 cycles of 15 s at 95ºC and 1 min at 60ºC. All data was normalized to the gene expression of β-actin. Fold change and standard deviations were calculated using the ∆∆CT method.

**Table 1:** Murine genes of interest and the exon targeted by the TaqMan mRNA assay probe

Gene	Receptor	Assay ID	Exon
*Vipr2*	VPAC2	Mm.PT.58.12387743	8–9
*Vipr1*	VPAC1	Mm.PT.58.42984475	4–6
*ADCYAP1R1*	PAC1	Mm.PT.58.32048683	9–10
*ACTB*	β-actin	Mm.PT.39a.22,214,843.g	5–6

### Statistical analyses

Briefly, mice were randomized with stratification according to home cage, sex, treatment, and 50% withdrawal thresholds measured at baseline day 1. Treatment groups and sex were equally divided throughout the test day. Group sizes were based on our previous work with these models where 12 animals per group produced sufficient power to detect intermediate size effects [16, 18].

The primary outcome was the difference in area under the curve (AUC) for 50% withdrawal thresholds between the two groups (WT and KO mice) receiving PACAP38. We calculated AUC according to the trapezoidal rule on the difference from baseline to obtain a summary measure to analyze the differences in PACAP38 response between groups using two-tailed unpaired t-test. We chose the AUC measure as our primary test because experiments showed that VPAC1 KO mice did not have the same baseline cutaneous tactile thresholds as the WT mice. By this approach we were able to align our statistical approach between the three KO mouse strains. Also, this is the approach commonly employed in human provocation studies using migraine triggers, where AUC on baseline subtracted values serves as the primary measure for assessing differences between two groups [24, 25]. As secondary outcome we conducted analysis using two-way repeated measures ANOVA to assess differences between groups in the experiments on each test day. Subsequent Tukey’s *post-hoc* test was performed comparing all groups. Rotarod data were analyzed by Kruskal-Wallis one-way ANOVA with Dunn’s post hoc comparison Myograph data are shown as mean ± SD, whereas qPCR data are shown as fold change with geometric mean ± SD. Significance levels in figures are shown as: * = *p* < 0.05, ** = *p* < 0.01, *** = *p* < 0.001, and **** = *p* < 0.0001. In myograph studies, unpaired t-test with Welch’s correction was used. qPCR results were compared using Student’s t test. *P* < 0.05 was considered statistically significant. All statistical analyses and graphs were done in GraphPad Prism 9 (Graph Pad Software Inc., CA, USA).

## Results

We administered PACAP38 to three knockout (KO) mouse strains lacking the PACAP-receptors VPAC1, VPAC2, and PAC1, respectively, and compared to their WT controls. The mice ranged in age from 8 to 18 weeks. The animals were carefully randomized to ensure that there were no age differences between the experimental groups within each strain. No adverse effects were observed following treatment with PACAP38, and all mice had normal stools and stable weights. Motor function of WT mice and KO mice was the same and was consistent with what we have observed previously for WT mice [17] We also looked at the potential sex differences, but the sample size in the current study was too small to see any meaningful differences and there were no trends towards sex differences in the data.

### VPAC1 KO strain

**Fig. 2:**
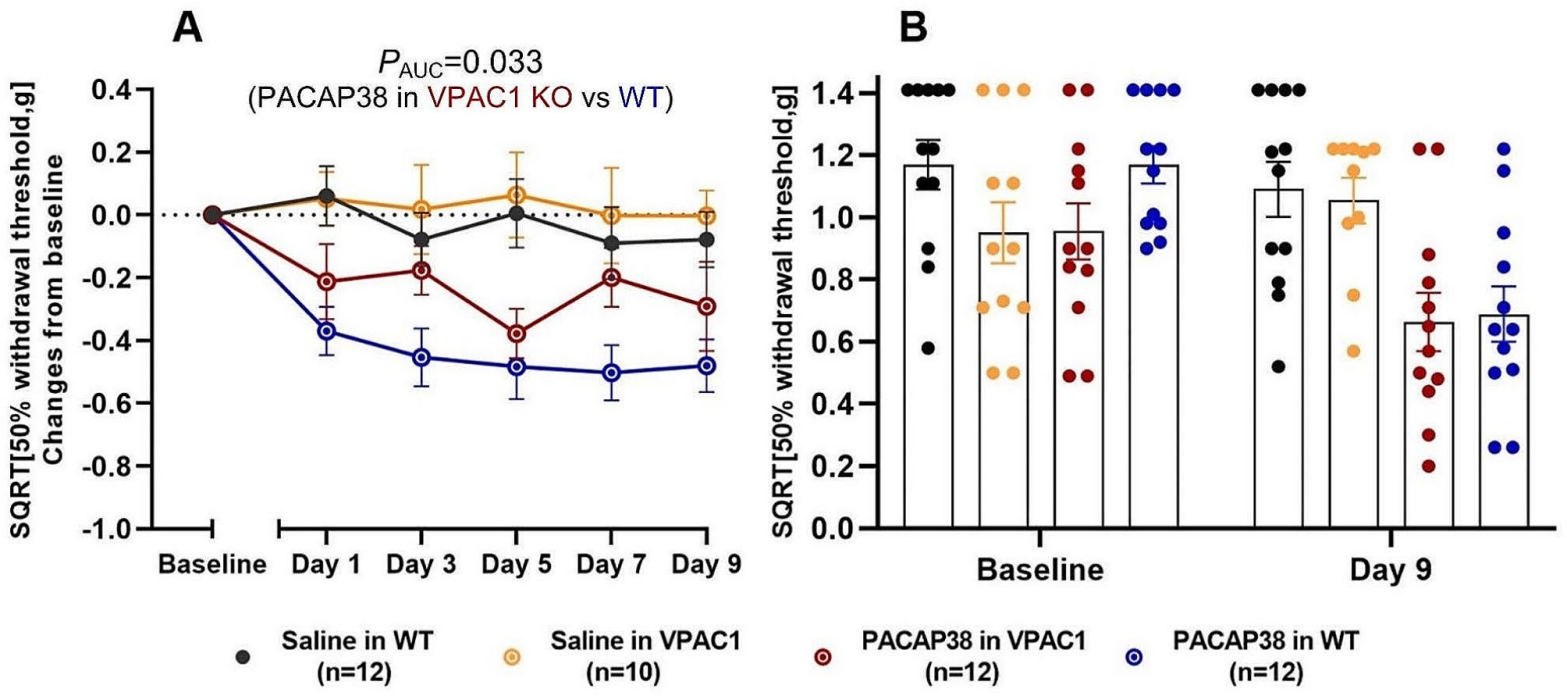
PACAP38 caused less hypersensitivity in VPAC1 KO mice (red line) than in WT (blue line). Concurrently, PACAP38 induced migraine-relevant hypersensitivity in both WT and VPAC1 KO mice when compared to saline controls (*p* < 0.01). (**A**) Responses one hour after subcutaneous administration of PACAP38 (2 g/kg) or saline to VPAC1 KO mice on five test days. (**B**) Descriptive representation of individual absolute data points for baseline and day 9 (acute response) with mean bars and SEMs

The mean weight at baseline of the VPAC1 KO was significantly lower than the WT mice (17.6 ± 0.5 g vs. 24.7 ± 0.4 g, *p* < 0.001). We found that PACAP38 induced less cutaneous hypersensitivity in VPAC1 KO mice than in WT mice measured as change from baseline using AUC (*p*_AUC_ = 0.033) (Fig. 2A). This suggests that the VPAC1 receptor plays a role in mediating PACAP38-induced hypersensitivity. Yet, both WT and VPAC1 KO mice displayed hypersensitivity after PACAP38 when compared to saline controls (*p* < 0.01). A significant difference between the two WT groups, that received either PACAP38 or saline, was found on all test days at the 1 h time point *(p <* 0.01 to 0.0001*)* (Fig. 2A). We only found a significant difference between the two VPAC1 KO groups on day 5 at the 1 h time point (*p =* 0.005). We did not find any difference between the two saline control groups (WT and KO) of withdrawal thresholds on day 9 (acute response) from baseline *(p* > 0.05). Moreover, basal thresholds (daily test prior to injections) on day 5,7 and 9 (*p* < 0.01 to 0.001, data not shown) were significantly decreased after PACAP38 as compared to saline controls. After PACAP38 administration, VPAC1 KO mice had a decrease in mean SQRT 50% withdrawal threshold from 0.96 ± 0.09 g to 0.66 ± 0.09 g (0.30 g difference) when comparing baseline and day 9, whereas WT mice had a decrease from 1.17 ± 0.06 g to 0.69 ± 0.09 g (0.48 g difference). Notably, the mean SQRT 50% cutaneous thresholds at baseline were lower for VPAC1 KO mice compared to WT mice (0.96 ± 0.09 g vs. 1.17 ± 0.06 g, *p* = 0.010) (Fig. 2B).

### VPAC2 KO strain

**Fig. 3:**
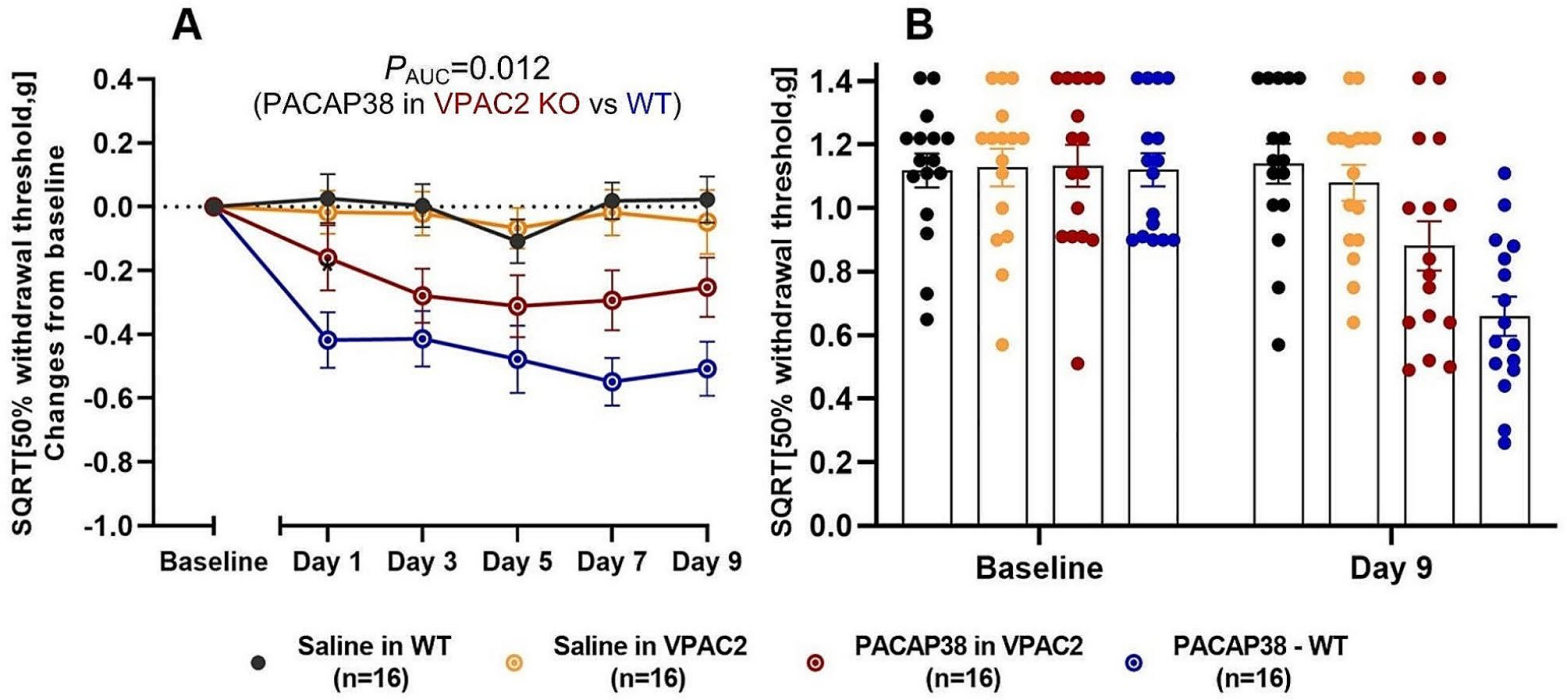
PACAP38 induced less hypersensitivity in VPAC2 KO mice (red line) than in WT (blue line). Concurrently, PACAP38 induced migraine-relevant hypersensitivity in both WT and VPAC2 KO mice when compared to saline controls (*P* < 0.0001). (**A**) Responses one hour after subcutaneous administration of PACAP38 (2 g/kg) or saline to VPAC2 KO on five test days. (**B**) Descriptive representation of individual absolute data points for baseline and day 9 (acute response) with mean bars and SEMs

The VPAC2 KO and WT mice showed no difference in mean weight at baseline (25.2 ± 0.5 g vs. 25.8 ± 0.6 g, *p* = 0.409). As in the VPAC1 KO mice, PACAP38 induced less hypersensitivity in VPAC2 KO mice than in WT mice (*p*_AUC_ = 0.012) (Fig. 3A). This suggest that the VPAC2 receptor also plays a role in mediating PACAP38-induced hypersensitivity. Both the WT and VPAC2 KO mice had decreased withdrawal thresholds after PACAP38 injection when compared to saline controls (*p* < 0.0001). A significant difference between the two saline groups and the PACAP38 WT group was found on all test days at the 1 h time point *(p <* 0.01 to 0.0001*)* (Fig. 3A). We only found a significant difference between the two VPAC2 KO groups on day 3 and 7 at the 1 h time point (*p =* 0.046 and *p =* 0.029). In addition, the two saline control groups (WT and KO) showed no difference in withdrawal responses (*p* > 0.05). After PACAP38 administration, VPAC2 KO mice had a decrease in mean SQRT 50% withdrawal threshold from 1.13 ± 0.07 g to 0.88 ± 0.08 g (0.25 g difference) when comparing baseline and day 9, whereas WT mice had a decrease from 1.12 ± 0.05 g to 0.61 ± 0.06 g (0.51 g difference). Direct comparison of absolute SQRT 50% thresholds between VPAC2 KO and WT on day 9 revealed only just no statistical difference in response (*p* = 0.052) (Fig. 3B).

### PAC1 KO strain

**Fig. 4:**
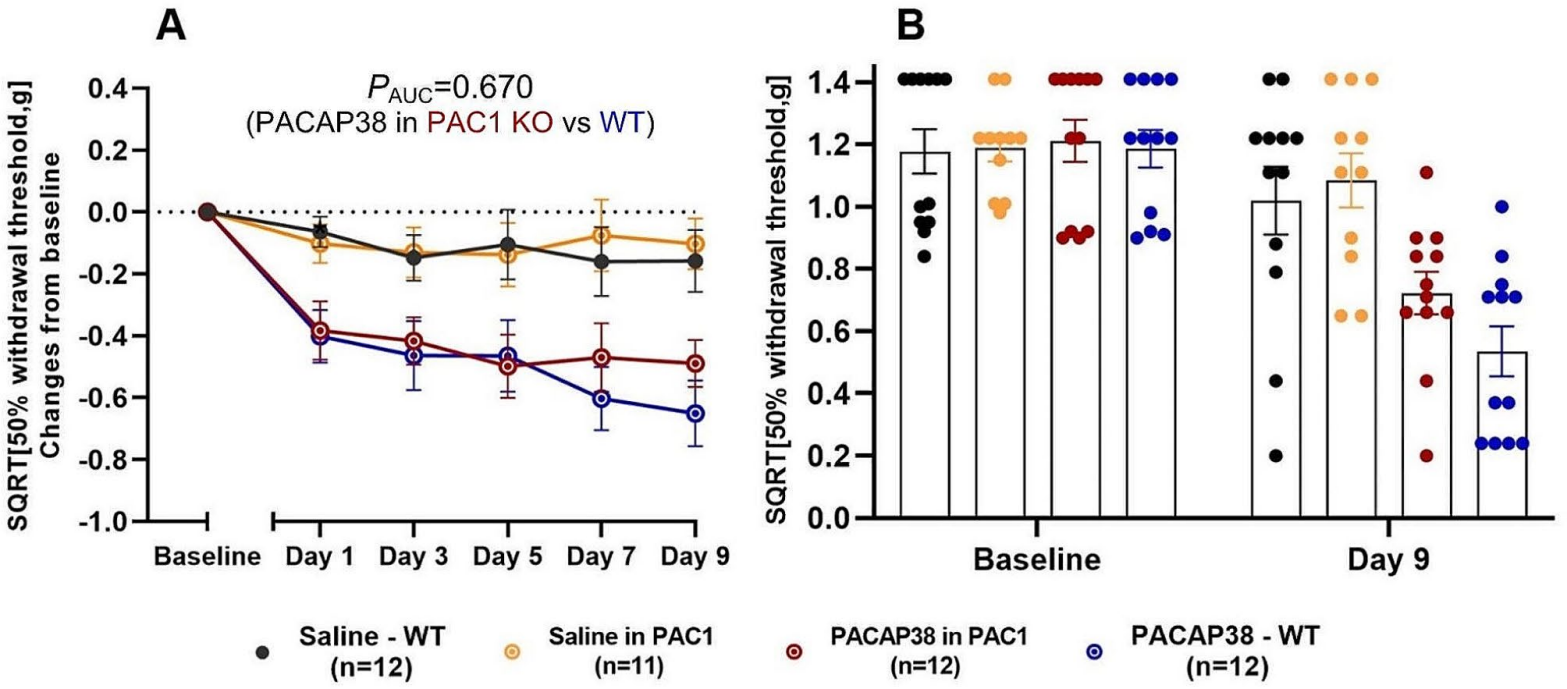
PACAP38 induced significant hypersensitivity in both WT and PAC1 KO mice (*p* < 0.0001), but we found no significant difference in PACAP38 response between WT and PAC1 KO mice. (**A**) Responses one hour after subcutaneous administration of PACAP38 (2 g/kg) or saline to PAC1 KO mice on five test days. (**B**) Descriptive representation of individual absolute data points for baseline and day 9 (acute response) with mean bars and SEMs

The PAC1 KO and WT mice showed no difference in mean weight at baseline (25.3 ± 0.5 g vs. 25.7 ± 0.5 g, *p* = 0.150). For PAC1 KO mice, we observed no difference in the response to PACAP38 compared to the WT (*p* = 0.670) (Fig. 4A). Both WT and PAC1 KO mice displayed decreased thresholds after PACAP38 injection when compared to vehicle controls (*p* < 0.0001). A significant difference between the positive and negative controls, receiving PACAP38 and saline, respectively, were found on all test days at the 1 h time point (*p* < 0.01 to 0.0001). After PACAP38 administration, PAC1 KO mice had a decrease in mean SQRT 50% withdrawal threshold from 1.21 ± 0.6 g to 0.72 ± 0.6 g (0.49 g difference) when comparing baseline and day 9, whereas WT mice had a decrease from 1.19 ± 0.6 g to 0.54 ± 0.7 g (0.65 g difference). PAC1 KO and WT responses to PACAP38 on Day 9 were not significantly different (Fig. 4B).

### Motor function

To ensure that the von Frey tests were not biased by impaired motor coordination, the rotarod test was performed on all mice at baseline and after PACAP38 injection (day 9). Motor function on the rotarod in all KO mice were the same as WT mice at baseline. The motor functions were unaffected after PACAP38 administration in both KO mice and WT mice and showed no difference between any of the tested groups (*p* > 0.05; data not shown).

### Myograph

**Fig. 5:**
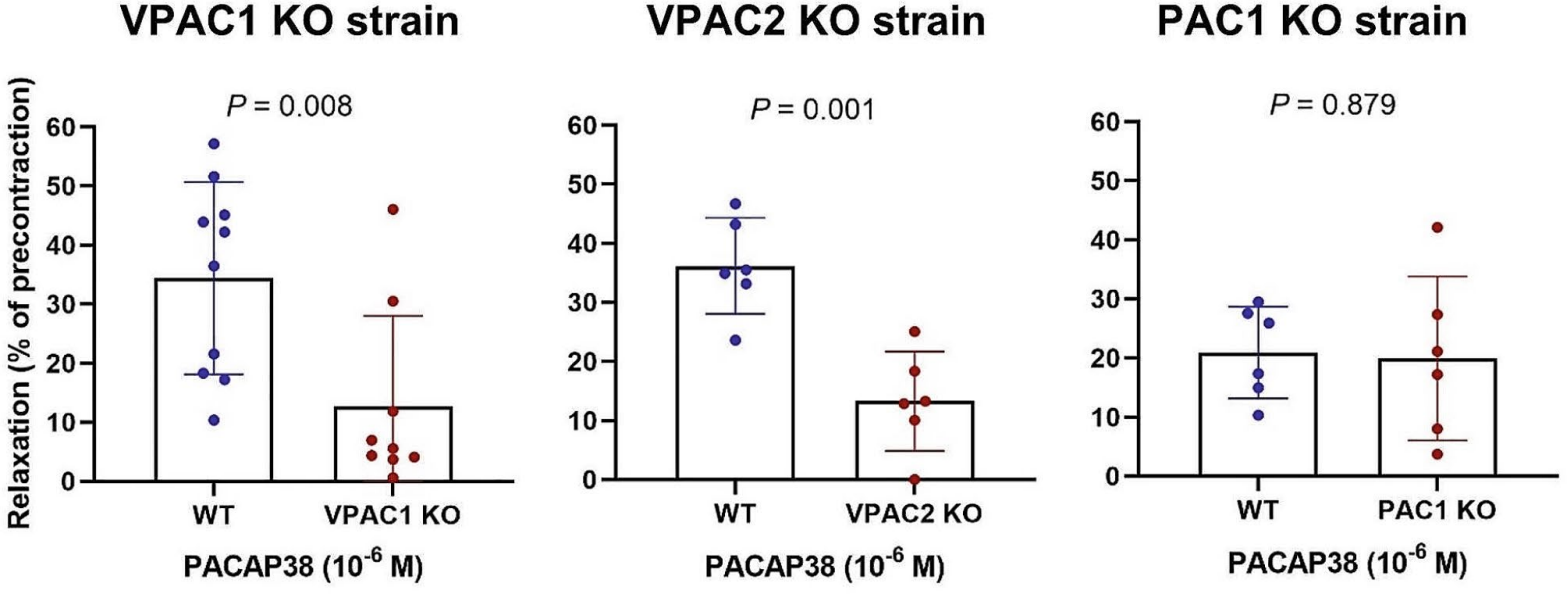
Myograph studies comparing the response to PACAP38 of the common carotid artery of 10 VPAC1 KO, 6 VPAC2 KO and 6 PAC1 KO mice and their WT control mice. After precontraction (0.03 µM U46619), arteries were exposed to 1 µM PACAP38. Data are presented as mean ± SD. Student’s unpaired t-test was used, each dot representing artery segment from individual mice

The common carotid artery from VPAC1-, VPAC2-, PAC1 KO mice and their WT controls were exposed to PACAP38 (Fig. 5). PACAP38 induced significantly less relaxation of the artery in VPAC1 and VPAC2 KO mice as compared to WT. In the VPAC1 KO mice, PACAP38 induced a relaxation of 12.7 ± 15.3% of the precontraction, whereas the corresponding response in WT mice was 34.4 ± 16.3% of the precontraction (*p* = 0.008). In the VPAC2 KO mice, PACAP38 induced a relaxation of 13.3 ± 8.4% of the precontraction, whereas the WT mice exhibited a relaxation of 36.2 ± 8.1% of the precontraction (*p* = 0.001). In the PAC1 KO mice, PACAP38 induced a relaxation of 20.9 ± 7.8% of the precontraction, whereas the WT mice exhibited a relaxation of 19.9 ± 13.9% of the precontraction (*p* = 0.879). Thus, the PAC1 KO mice and their WT controls showed no difference in vasoactivity after PACAP38. Notably, the WT controls of PAC1 KO mice had seemingly a lower response to PACAP38 than the WT of the other strains.

### qPCR

**Fig. 6:**
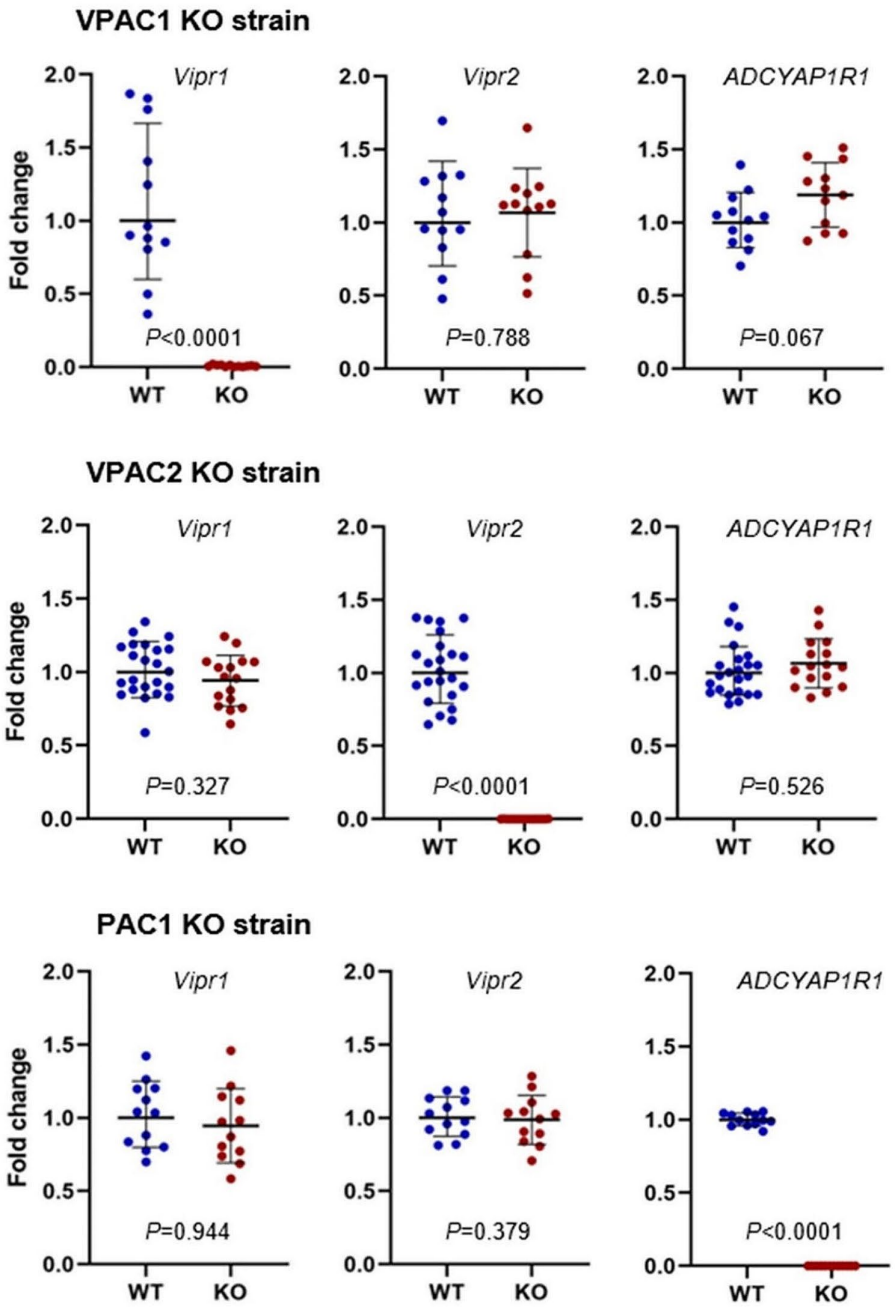
mRNA levels of PACAP38 receptors in TNC for VPAC1, VPAC2 and PAC1 KO mice strains vs. WT. We found no differences between KO mice and WT mice regarding mRNA expression of the non-modified PACAP receptors in any of the three strains. Results are presented as individual points of mRNA expression fold change with geometric mean ± SD. WT is set to 1 and results are normalized to the levels of β-actin. Statistics are calculated from individual ΔCt (delta cycle threshold)

To investigate if any compensatory upregulation of the non-modified PACAP receptors had occurred in the KO mice compared to WT, we performed qPCR to quantify gene expression of the three PACAP receptor genes *Vipr2*, *Vipr1*, and *ADCYAP1R* in the TNC. We found no differences between KO mice and WT mice regarding mRNA expression of the non-modified PACAP receptors. Deletion of the target receptor was confirmed at the transcriptomic level for all strains (Fig. 6).

Because VPAC2 KO mice was easiest to breed, we initially investigated mRNA levels of the PACAP receptors in the TG, carotid artery, dura mater, and TNC in 16 VPAC2 KO mice and 23 WT mice. The data showed that expression of *vipr1* and *vipr2* mRNA in the TG, carotid artery, and dura mater were either extremely low or undetectable (CT > 35) in both WT and VPAC2 KO mice using our method, preventing further analyses. This is the reason that we subsequently only performed qPCR on tissues from TNC in VPAC1 and PAC1 KO mice versus their WT controls.

The mice in each group were both males and females and PACAP38-treated and saline-treated, but we found no differences in mRNA levels between treatment and sex for the non-modified receptors in all three strains (*p >* 0.05) (data not shown). Hence, we have merged the groups so a total of 12 KO mice and 12 WT mice were examined for the VPAC1 and PAC1 KO strain.

## Discussion

We investigated PACAP38-induced hindpaw hypersensitivity as a mouse model of migraine. Our data showed that VPAC1 and VPAC2 KO mice displayed reduced PACAP38-responses compared to WT, whereas PAC1 KO mice showed no difference to WT. The reduced PACAP38-responses in VPAC1 and VPAC2 KO mice seem to be due to partial inhibition as the response is not as strong as the control group receiving saline. These results indicate that VPAC1 and VPAC2 receptors might together, rather than individually, contribute to migraine-relevant hypersensitivity in a validated mechanistic model. These results are supported by our myograph data that showed diminished vasoactivity in VPAC1 and VPAC2 KO compared to WT mice in response to PACAP38, but no difference was found between PAC1 KO and WT arteries. qPCR showed no compensatory upregulation in gene expression of the non-modified PACAP receptors in KO mice. These results indicate that PACAP38 likely exerts its migraine-inducing action by effects on both of the VPAC-receptors.

### Which receptors are involved?

Our current results showing that VPAC1 and VPAC2 receptors might contribute to migraine induction is consistent with the observation that prolonged VIP infusion can also trigger migraine episodes in adults with migraine [11]. Moreover, a mAb inhibiting the PAC1 receptor, did not demonstrate any therapeutic benefit over placebo in a phase II clinical trial [26]. Despite these findings in humans, it is debated whether the PAC1 receptor or the VPAC receptors are involved in migraine or other primary headaches. Pharmacological inhibition of the PAC1 receptor has been shown to prevent GTN-induced hypersensitivity in mice [27] and a rodent specific PAC1 antibody was able to inhibit evoked nociceptive activity in rats [28]. Additionally, a previous study showed that inhibition of PAC1 receptor internalization improved mechanical hypersensitivity induced by repeated administration of PACAP38 in rats [29]. Another study using PACAP receptor antagonists, found that VPAC1 and PAC1 receptors regulate lacrimal blood flow and trigeminal neuron activation, while VPAC2 does not [30]. This contrasts with our findings, but an important difference is that they used VIP [6–28] as a non-selective VPAC2 antagonist, PG-97-269 as a selective VPAC1 antagonist, and the non-selective PAC1 antagonist, PACAP- [6–38], which also is a strong agonist on the MrgB3 receptor [31]. The differences in tools used preclude conclusive comparison with our observations. Taken together, our results, along with those from human studies, corroborate the notion that VPAC1 and VPAC2 are the primary PACAP receptors implicated in migraine induction.

### Speculations on mast cell degranulation

There is increasing evidence for a role of PACAP signalling in mast cell degranulation. For example, There PACAP38 is known to activate a fourth PACAP receptor on mast cells [31] called MrgB3 in rats, MrgB2 in mice, and MrgX2 in human. The effect of PACAP38 on MrgB3-induced mast cell degranulation has previously been shown in rats [31]. Furthermore, its receptors, VPAC1 and VPAC2, may also mediate mast cell degranulation [32, 33].

In humans, intravenous administration of PACAP38 induces significant skin flushing, a phenomenon mitigated by sumatriptan [34]. Intradermal injection of PACAP38 induces a localized increase in blood flow, swelling and redness of the skin in healthy volunteers [35]. Although, pretreatment with H_1_-antihistamine, clemastine, did not prevent PACAP38-induced migraine or flushing [36] which speaks against a mediating role of histamine, mast cells do secrete a wide range of other inflammatory mediators [37]. In patients with inflammatory bowel disease (IBD) VPAC1 is upregulated in mucosal mast cells. Thus, upregulation is possible in cells that normally may not express VPAC1 [38]. It is of note that IBD and migraine are co-morbid [39]. VPAC2 receptors are expressed in human mast cells and are essential in immunological and inflammatory diseases [32, 40].

A recent study using genetically modified mice lacking the Mrg receptor found that MrgX2 KO mice had partially reduced PACAP38-induced facial hypersensitivity compared to WT expressing the human MrgX2 receptor, which supports the notion that other receptor subtypes contribute to the pro-nociceptive effects of PACAP38 [41]. The mouse MrgB2 receptor was expressed on meningeal connective tissue mast cells and contributed to PACAP38-induced migraine-relevant hypersensitivity. In various animal models, mast cell degranulation was observed in the dura mater following PACAP38 exposure [33, 42]. These data, along with our findings, support that mast cell degranulation may be an important mediator of the migraine-inducing effect of PACAP38.

### Arterial dilation

We showed that VPAC1 and VPAC2 receptors are the main mediators of arterial dilation induced by PACAP38. For unknown reasons, the WT controls of the PAC1 strain exhibited a lower response to PACAP38 compared to the WT of the other strains. All experimental procedures were identical and balanced across sex, age, and WT/KO but experiments on the three strains were separate in time allowing for random effects to unevenly influence the responses. Thus, the comparison between KO and WT within each strain is therefore more valid than the comparisons between strains. Our results align with prior in vivo and ex vivo studies, indicating that vasodilation induced by PACAP38 and VIP is facilitated through VPAC1 and VPAC2 receptors [43, 44]. All known compound triggers of migraine exhibit vasodilatory characteristics, suggesting that vasodilation of cephalic arteries might serve as a partial proxy for migraine pain [25]. In humans, intravenous PACAP38 (10 pmol/kg/min) and VIP (8 pmol/kg/min) are seemingly equally potent vasodilators of the middle meningeal and superficial temporal, but none of the peptides dilate the middle cerebral artery [45]. The vasodilatory effect of PACAP38 is sustained compared to VIP and may be due to activation of Mrg receptors and mast cell degranulation by PACAP38, leading to potentiation of the effect. In rats, the long-lasting dilation of the middle meningeal artery characteristic of PACAP38 was diminished by both, prior anti-histamine treatment and mast cell depletion [46]. PACAP38 is a stronger degranulator than VIP with pEC50 values of 6.58 whereas the pEC50 for VIP is between 5.49 and 4.78 [33]. This may be reflected clinically where PACAP38 is a more potent migraine inducer than VIP [45]. Nonetheless, VIP may also be a potential drug target as migraine treatment. Overall, these studies suggest that release of inflammatory mediators from mast cells contribute to meningeal arterial dilation following PACAP38 and VIP infusion, but a synergistic effect likely exists regarding vasodilation and mast cell degranulation.

### Strengths and limitations

We originally planned to include the Mrg receptor in our study, but we were not able to make, find or acquire MrgB2 deficient mice. It would have been elegant to test all four known PACAP receptors under the exact same experimental conditions. Regarding the KO strains, it is well known that the VPAC1 KO strain displays abnormal intestinal development and weighs significantly less than WT controls [13] and we found that cutaneous tactile thresholds in KO were lower than WT at baseline. The difference in weight may have influenced blinding of KO and WT mice, but blinding for treatment with PACAP38 or saline was unaffected. All three strains of KO mice showed intact motor coordination, supporting the validity of our measurements. PACAP38 was administered subcutaneously in mice, differing from i.v. infusions in humans [3, 24]. It is possible that our KO mouse strains exerted compensatory mechanisms that might have obscured the actual effect of PACAP38-induced hypersensitivity and vasodilation on the PACAP receptors. It may therefore be relevant to confirm our findings using selective chemical inhibitors of the receptors whenever available. In addition, genetic variations in PACAP and its receptors, resulting from gene splicing, can significantly affect the selectivity, potency, and signaling of PACAP receptors [47]. Yet, in our study, we did not detect any regulation at mRNA level of the non-modified PACAP receptors in KO mice that could be suggestive of compensatory mechanisms. We did not do experiments in heterozygous mice or VPAC1 + VPAC2 double KO. In the former, the effect size would likely be small and require unreasonable large sample sizes. The latter may be done in future studies but mouse reproduction and/or health could be severely affected.

While we acknowledge that others have used periorbital sensitivity measurements for their migraine studies [48], we have focused on plantar tactile sensitivity as primary readout for the current study. We reason that there is accrued evidence that both plantar and periorbital sensitivities are equally relevant in migraine research, as increased cutaneous mechanical sensitivity can be induced by GTN, levcromakalim, cilostazol and PACAP38 in both anatomical areas [6, 48]. Also, measurement of periorbital sensitivity typically shows higher variability and a narrower effect window compared to the plantar region, demanding larger experimental groups and reducing 3R compliance [49]. Lastly, as discussed in our previous publication [6] both hypersensitivities can be inhibited by migraine-specific drugs without general analgesic effects [50, 51].

## Conclusion

The present study assessed the role of three PACAP-receptors in a single in vivo mechanistic migraine mouse model. The findings suggest that VPAC1 and VPAC2 receptors may both be involved in mediating migraine-relevant pain. Therefore, solely targeting specific PACAP receptors might not be effective for migraine treatment, whereas focusing on the circulating PACAP ligand, mast cells, or circulating VIP may offer more promising targets for migraine therapy.

## Data Availability

The datasets used or analyzed during the current study are available from the corresponding author on reasonable request.
